# The role of the media on maternal confidence in provider HPV recommendation

**DOI:** 10.1186/s12889-020-09877-x

**Published:** 2020-11-23

**Authors:** Kimberly K. Walker, Heather Owens, Gregory Zimet

**Affiliations:** 1 Zimmerman School of Advertising and Mass Communications, University of South Florida, 4202 E Fowler Avenue, CIS1040, Tampa, FL 33620 USA; 2 College of Public Health, University of South Florida, Tampa, USA; 3 Department of Pediatrics, Indiana University School of Medicine, Indianapolis, USA

**Keywords:** Patient/provider relationship, Human papillomavirus, media, provider recommendation, Qualitative, Communication

## Abstract

**Background:**

Despite a growing understanding of the importance of provider HPV recommendation on parental acceptance, U.S. HPV vaccination rates remain suboptimal. Given the prevalence and use of the media for health decisions, this study examined the relationship between the media and provider HPV recommendation on maternal HPV vaccine hesitancy.

**Methods:**

Thirty individual interviews with HPV vaccine-accepting mothers in the Midwest U.S. were conducted to examine their feelings of hesitancy around the decision to accept HPV vaccination at the time of provider recommendation and their suggestions for improving the recommendation experience by addressing media concerns.

**Results:**

Media exposure was an antecedent to hesitancy for three main vaccination concerns: safety, protection/efficacy and sexual stigma. Although mothers accepted vaccination, they continued to feel confused and hesitant about HPV vaccination. They had several recommendations for how providers could combat hesitancy to improve confidence in HPV vaccine acceptance.

**Conclusions:**

Providers’ approach to HPV vaccination recommendation must consider concerns reported in the media with delivery techniques modified to adjust to maternal fears absorbed from adverse media information.

**Supplementary Information:**

The online version contains supplementary material available at 10.1186/s12889-020-09877-x.

It is well known that the human papillomavirus (HPV) causes nearly all cervical cancers and many cancers of the vagina, vulva, penis, anus, and oropharynx [1]. In the U.S., HPV causes nearly 36,000 cancers each year [2]. In 2006, the FDA approved a quadrivalent vaccine as a primary preventive strategy to reduce HPV infections and diseases [2]. The 9-valent vaccine, which is the only HPV vaccine currently available in the U.S., has the potential to prevent the majority of HPV-related cancers [3]. However, despite strong national recommendation to vaccinate adolescents against HPV, uptake has been suboptimal compared to other routine vaccinations in adolescents. As of 2019, HPV vaccine initiation and completion rates in the U.S. were only 71.5 and 54.2%, respectively [4].

The poor uptake rate of the HPV vaccine has been attributed to many factors, of which parental hesitancy is one of the strongest [5]. Many studies have documented high rates of parental hesitancy around HPV vaccination, contributing to delay of vaccination or vaccine refusal [6–8]. According to a national survey, at least one-third of families are vaccine hesitant [9]. Given the fact that many of the vaccine-preventable diseases are no longer as prevalent as they once were, parents are often not aware of the devastating effects these diseases can have on their children’s health; therefore, understanding sources of parental hesitancy is important for developing strategies to address their concerns [5, 9, 10].

Low coverage rates have prompted considerable research on the determinants of HPV vaccination. The primary influence on parental HPV decisions highlighted in the literature is the healthcare providers’ recommendation; those who receive a provider recommendation are more likely to vaccinate their children than those who do not [6, 11]. Yet, the fact that a parent chooses vaccination does not necessarily mean that he/she is confident in the vaccination decision. Hesitancy exists along a continuum of indecision to include individuals who are neither strongly pro- nor anti-vaccine [12–14]. Vaccine-hesitant parents may accept certain vaccines, refuse others, delay initiation, or accept but feel unsure about doing so [12–14]. Several studies show that educated parents may be more likely to experience hesitation upon recommendation than less educated parents [15–17]. One theory behind this observation is that educated parents are more likely to have access to specific sources of the media, such as the internet, which may expose them to contradictory information about vaccines, including HPV vaccine [5, 18, 19]. Additionally, educated parents may feel more confident in their ability to interpret complex scientific and clinical health information, which may allow them to ignore their provider’s advice if contradictions exist [5]. Research has found that parents who do not have their children vaccinated often have researched the topic extensively [5, 20].

Misinformation and hesitancy among HPV vaccine accepting parents are largely unexplored. In today’s health conscious society, seeking information about health topics is increasing, with one in three U.S. adults using the internet to self-diagnose or learn about a health concern [21]. The growth in internet use and increase in health information available on the web has changed the landscape of health information [21]. At one time, medical knowledge was almost entirely disseminated by health professionals. Now, anyone can share medical content, both accurate and inaccurate, that is accessed via web-based searches. In addition to active information-seeking, the media’s ubiquitous presence in everyday lives presents opportunities for passive exposure to varying and often conflicting opinions on health behaviors, including vaccinations. Given the presence and potential influence of the media on HPV vaccination attitudes [22], more exploration is needed on the ways in which media exposure to HPV vaccination information may affect parental decisions about provider HPV recommendations [22].

The study purpose was to qualitatively explore the complex relationship between provider HPV vaccination recommendation, media, and vaccine hesitancy among a group of educated mothers who had vaccinated a child against HPV but remained hesitant. Mothers’ perspectives were also elicited on ways to improve the provider/patient HPV recommendation experience in light of the current media environment.

## Methods

### Procedure

The approach used in this study was based on the social-ecological model and the health belief model (HBM). Social-ecological systems theory is a model that underscores how characteristics of the environment influence individual health behavior and outcomes, while the HBM is a social psychological health behavior change model developed to explain and predict health-related behaviors, particularly in regard to the uptake of health services such as vaccination [23, 24]. A previous analysis of these data focused on the wide range of socio-ecological influences of HPV vaccine hesitancy among mothers who had chosen to vaccinate their children (accepted and forthcoming). Because all mothers discussed the media in light of their decisions, the present study focused solely on the socio-ecological influence of the media on hesitation, within the context of a provider’s recommendation. The HBM construct of perceived barriers was used to frame how mothers’ use of media drove their hesitancy about HPV vaccination. The HBM construct of cues to action was used to understand the strategies that mothers believed could help to overcome negative media messages about the HPV vaccine.

Thirty mothers from the Midwest U.S. were recruited for this study. Only mothers were included because research shows that they are most often the primary vaccination decision-makers for their children or share this role with a parenting partner [25]. Mothers were eligible to participate if they had obtained a bachelor’s degree or higher and had at least one child 9–18 years old who had been vaccinated for HPV. The sample was obtained through a combination of purposive and snowball sampling. The lead author initially approached mothers affiliated with a parent advisory group associated with a midwestern pediatric medical department, via email, and invited them to participate. These mothers then recruited other mothers to join. Participants were told the study purpose was to understand their HPV vaccination decisional processes to guide and empower other mothers to vaccinate their children. The study protocol was reviewed by the University of South Florida’s (USF) IRB and granted exempt status (pro00041072). The requirement for informed consent was waived.

### Data collection

A semi-structured questionnaire interview guide was developed specifically for this study. It included open-ended questions to elicit discussion about mothers’ decisions about HPV vaccination and was developed by the research team, which was comprised of researchers in the fields of adolescent health psychology, health communication and epidemiology. The original guide was developed to explore the possibility of any social-ecological level influence on mothers’ HPV vaccine hesitancy. The interview began with general questions about mothers’ experiences with the HPV vaccine. Mothers were then asked to recall what they remembered of their experiences and challenges when considering the provider’s recommendation for HPV vaccination and to relay how those experiences shaped what they believe could improve the patient/provider experience with the recommendation. As media influence was brought up by all participants, probing questions addressing the impact of the media were included as the focus of the present study. Specifically, the goals of the current study were to 1) examine media influence as a determinant of decision-making at the time of a provider recommendation and 2) describe mothers’ recommended strategies to cue improved provider recommendations for HPV vaccination within the context of media influences on health seeking and beliefs. The guide was subsequently pilot tested with mothers during preliminary interviews and revised in an iterative manner. Interviews were conducted over the telephone from September, 2019, to February, 2020, and lasted approximately 30 min each. Participants received a $25 gift card to compensate them for the time and effort required for their participation.

### Data analysis

Interviews were audio-recorded and transcribed verbatim, and thematic content analysis using an established qualitative approach was applied to identify themes and patterns within the data [26, 27]. Multiple readings of the interview transcripts were conducted by the research team (KW, HO, GZ) and subsequently discussed. Two of the researchers (KW and HO) then engaged in open coding to identify and label any component that related to participants’ expressions of media as they related to hesitancy about HPV vaccination. In axial coding, the two researchers met to categorize the emergent data into themes that described the media, hesitancy and provider HPV recommendation experiences. Through detailed conversations across data analysis, both authors refined the specific descriptive codes within each theme, discussed any discrepancies, and reached consensus regarding the consistency of the themes and codes. No new categories emerged after completing axial coding, which suggested theoretical saturation [28]. To comprehensively describe participants’ experiences, both authors integrated categories in selective coding to develop a stand-alone narrative reflected in the “Findings” section [29]. The lead author returned to the data once more to gather exemplar quotes and select excerpts to include, and composed the final report of the findings.

## Results

### Sample

Thirty mothers were recruited (See Table 1). They were ages 36–58 years. Nineteen identified as White, 10 as Black or African American, and 1 as Pacific Islander. Mothers had between 1 and 5 children, with half having 2 children. Forty-seven percent (*n* = 37) of children were females and 53% (*n* = 42) males.

**Table 1:** Demographics

	N(30) (%)
**Age**	
29–39	2 (6.67%)
40–49	18 (60.00%)
50–59	10 (33.33%)
**Race**	
White	19 (63.33%)
Black	10 (33.33%)
Pacific Islander	1 (3.34%)
**Number of Children**	
1	3 (10.00%)
2	15 (50.00%)
3	7 (23.33%)
4–6	5 (16.67%)
**Sex of Children**	
Female	37 (46.84%)
Male	42 (53.16%)

### Media as antecedent to hesitancy

Mothers reported having their children vaccinated by their family pediatrician at what was typically a well-child visit. All but 5 reported hesitancy upon provider recommendation, with 14 specifically mentioning a decision to delay vaccination. For instance: “*I was hesitant at first, I hadn’t thought about it yet. (Our) provider gave us the background, but I hadn’t had time to think about it”* (Participant 1)*.* Upon initial provider recommendation, all mothers said they had at least heard of the HPV vaccine but recalled negative messages in the media that they had read, seen or heard that contributed to hesitancy or factored into their decision to delay. Online internet exploration via search engines and posts from friends and family on online social media, primarily Facebook, were the primary sources of antecedent exposure. As example: *“There wasn’t a lot of research done then (about 4 years ago). He (provider) recommended it that year, and I just wasn’t ready for it yet. Of course, I had gone on the internet and read there were some side effects”* (Participant 2*).* Another mother told of hesitancy from online “stories” on social media: *“We waited until 12 or 13. Our kids are really active; I read stories that may or may not be true” (*Participant 3). Traditional media (defined as news, magazines and print sources) were mentioned less than online sources but were still recalled at the point of provider recommendation by a few. As one mother explained: *“I did have reservations; HPV (vaccine) hasn’t been out that long. You hear the pros and cons. … you always hear the worst case scenario on the news”* (Participant 4).

### Themes of concerns stemming from antecedent media exposure

Mothers who recalled media messages prior to provider HPV vaccine recommendation revealed three primary themes of hesitancy that stemmed from prior media exposure: safety/side effects, protection/efficacy, and sexual stigma.

#### Safety/side effects

Mothers’ narratives of hesitancy stemming from media messages about the HPV vaccine were foremost related to misinformation about adverse side effects from HPV vaccination, which they described as being widely communicated (albeit incorrectly), especially on the internet and on social media (e.g. Facebook). As one mother said: “*… there were parents I was reading and hearing about, saying that their kids had a reaction to the shot. That was alarming to me”* (Participant 2). The harms mothers spoke of reading about on social media and the internet were varied, ranging from paralysis to autism to general, vague claims of fevers, aches and pains. Most mothers stated they recognized that these claims of harm had no scientific basis, yet some still talked about postings from friends on Facebook that told of a child who became paralyzed or who immediately ran a fever and became sick following vaccination. These stories led some mothers to question whether the HPV vaccine may be harmful, especially if a child already has a health condition. Other than paralysis, most mothers dismissed extreme claims from anti-vaccine groups online.

Some mothers also questioned advertisements and commercials on television advocating HPV vaccination because they originated from a pharmaceutical company and “big business”. As a result, these mothers remained quite concerned about adverse effects. For example, “*There are so many advertisements you don’t know which ones are beneficial. How long has it been around? What are the long-term effects? What it does? What is it supposed to do? Are there negative contraindications?”* (Participant 5). Another mother stated, “*What are long-term effects of it? There is too much unknown. Knowledge is power for this. If I knew more about this—not from a pharmaceutical company, then I would be more apt (to get it for child)”*(Participant 6*).* One mother said that commercial advertisements were fear-inducing, making her uneasy about vaccine safety:*“The advertisement to me (that I remember) is the one with the kid away from school, has fever and can’t make go away. Whether they (media) are making attention, fear is out there. How safe it is was my main concern. It was definitely new with the oldest”* (Participant 7).

#### Protection/efficacy

As with safety, advertisements and commercials on television were also a source of hesitancy regarding the protective benefits of the vaccine. A couple of mothers spoke of distrust of the pharmaceutical-funded commercials, which resulted in questioning the efficacy of and need for the vaccine. For instance, one mother noted, “*It is media promotion. I think I would have been more secure without that promotion. The commercials – they flooded the networks*. *Honestly, I question it because the commercials are backed by drug companies”* (Participant 6). This mother differentiated that commercials are not the same as traditional news media and should not be trusted. Although commercials were the media most often mentioned in regard to distrust, mothers’ attention to negative media claims about the lack of data supporting long-term efficacy of a “relatively new vaccine”, which were gathered from multiple channels, including traditional and social media, had a couple of mothers wondering whether the HPV vaccine protects from any disease. A few mothers had either only read on the internet or on Facebook that the vaccine only protected from a sexually transmitted infection (STI) and were not aware of its cancer protective benefits, especially for males, at the time of recommendation. For instance, *I felt it (HPV vaccine)...from what I had read and knew at the time, was more of a protection for others. I feel it is not as much for him as his future spouse or other”(*Participant 8).

#### Sexual stigma

Mothers’ stories of their experience with provider HPV recommendation also uncovered that exposure to the internet and social media, particularly Facebook, led to concern and fear of sexual stigma related to the vaccine. Some mothers, as a result of what they had seen discussed on Facebook and online discussions, feared that the HPV vaccine was only for young people who were sexually active. As one mother concluded of the lack of necessity of the vaccine for her child, primarily from her online, internet readings:*“From what I have read, I think it (the vaccine) is (equated with being) sexually active. I knew my child was not”* (Participant 9). Mothers commonly recalled reading and following conversations from family and friends on Facebook that included strong opinion that allowing one’s child to be vaccinated for HPV would be equivalent to accepting and even encouraging the child to be sexually active. As one mother described of her hesitancy derived from online discussions: *“Even if you are leaning about wanting to do the right thing (vaccinate), the (sexual) stigma is there (on social media)* (Participant 10)*.* Also*,“There’s so much you read online. I was probably one of those initial ones who quickly attached it to sexual. And thought that (sex) wasn’t going to come now*” (Participant 3). Exposure to these online conversations about sexual outcomes was a source of hesitancy for some mothers because they did not want to attach stigma to their child or to themselves as a “bad” mother for agreeing to HPV vaccination. One mother described it this way: “*I just didn’t want this to be like my support of him having to jump out and have sex. I was concerned that is this giving him the green light to say yeah”* (Participant 11). A couple of these mothers indicated that online conversations about sexual promiscuity had them questioning why the vaccine was “suddenly” needed now (e.g. they wondered if there was a rise in STIs that would prompt need for the vaccine.)

### Decision-making from media exposure: risk benefit ratio and ongoing delay and hesitation

Mothers weighed the uncertainty of the vaccine’s protective effect, safety and sexual stigma gathered from traditional and online/social media sources against provider recommendation and were concerned about the risk of accepting vaccination. One mother described the struggle this way: “*You read about many more harms that can happen (by vaccinating) than good. Who doesn’t want to help protect their children? It’s more about fear”* (Participant 12). Mothers of sons and daughters were equally likely to delay, with a couple of mothers stating they had concerns with injecting a “foreign substance” without confidence of its protective impact. A couple of these mothers of sons delayed vaccination to discuss with their sons and allowed their sons to decide on vaccination.

Many mothers who delayed wanted to continue research about the vaccine on the internet. Most looked to sources of information such as WebMD and cancer organizations, but others referred more vaguely to online information seeking from unknown sources. Mothers also mentioned continuation of contact and discussions about HPV vaccination with friends on social media, especially Facebook. These mothers both described how they were exposed to the media prior to provider recommendations and also chose to actively use online information sources after recommendations to address anxiety: *“I took some time to think about it (HPV vaccination recommendation); I read up on it on my own. I always feel there is some information they don’t have insight to on commercials. I wanted the medical background. I had to come back for another appointment to have them vaccinated*” (Participant 13). Also,*“Before vaccinating my 14-year-old, I asked providers I worked with as friends. Providers said they absolutely would vaccinate. I also read information online”* (Participant 14).

A few mothers who had vaccinated an older child still needed reassurance by seeking information online themselves before vaccinating a younger child. For instance: *“I still have reservations about my (younger) daughter. I need to do more follow up online, to look at the stats--- what is the data showing now? Does it make a difference for kids getting the shots? I want the data. My daughter goes to the doctor today. I am going to be pressured again”* (Participant 2).

### Mothers’ provider recommendations for countering inaccurate media messages

Mothers indicated that what they had heard, read or seen from advertisements/commercials on television, the internet and social media negatively affected their acceptance of their provider’s HPV recommendation. Hesitation often occurred even when mothers stated they had good relationships with and trusted their provider. Based upon their own experiences with hesitancy from negative media messages, mothers most commonly believed providers could do more to counteract negative media messages so that other mothers might be more likely to accept the HPV vaccine recommendation. Less frequently, mothers also recommended the continuation of provider strategies that helped them to overcome their hesitancies and accept vaccination. The strategies mothers recommended pertained to the timing, delivery and content of the HPV vaccine recommendation in the three areas of safety, efficacy/protection and sexual stigma and are discussed next.

First, to counteract inaccurate media messages concerning safety, mothers recommended that providers emphasize the statistics surrounding the safety of the vaccine, including how many people have been vaccinated, the year it became available, the studies that support minimal side effects, and the type of testing the vaccine underwent before recommendation. Mothers suggested that providers be prepared with verbal and written messages, primarily in the form of brochures, to counteract the inaccurate, negative messages about adverse side effects reported in the media, especially those related to autism, paralysis and general, vague side effects that proliferate online. One mother described how her physician’s ability to counteract these negative messages influenced her acceptance and recommended that the strategy continue:*“I only did it (accepted vaccination) because I know and trust my physician so much and he could counter the negative messages about side effects I had seen and read”* (Participant 12).

To address efficacy and protection concerns from commercials and social media, mothers suggested physicians communicate HPV vaccine efficacy and need by telling how many people acquire HPV, the types of cancers the vaccine protects, reasoning behind the perceived rise in need for the vaccine, and studies of long-term efficacy. Some mothers recommended that providers emphasize that the vaccine provides long-term cancer protective benefits, given online questioning and distrust of HPV vaccine commercials produced by a pharmaceutical company. Mothers also suggested providers use language that emphasizes cancer prevention over the fear messaging that non-vaccination could leave the child vulnerable to cancer, due to their belief that “everything seen on TV seems to cause cancer.” On the other hand, some mothers described how their provider’s communication about supporting vaccination for their own family members helped them overcome their anxieties. As a result, they suggested providers communicate their own experiences with HPV vaccination (e.g., vaccination of children, grandchildren, nieces, nephews) to validate and counteract distrust and skepticism from commercials and online reports. For example, “*So, the HPV vaccine, when came up, I did have questions. I had seen on 20/20, Dateline about adverse effects. I was a little more skeptical of HPV than honestly any others because of medical. My question to my pediatrician was,“Did you give to your children?” When she said, yes, I said I am willing to do with my children”* (Participant 1).

To combat the stigma associated with the vaccine from misinformation on social media, mothers suggested that providers present the HPV vaccine in one of two framing contexts, either: 1) language that does not address the sexual transmission of HPV or the protection from an STI at all, but only as a protection from cancer or 2) directly, but sensitively, communicating HPV as a sexually transmitted virus alongside an approach that assures your child is not at “fault” but is being protected from a future partner, for which the child has no control over his/her sexual history. Both approaches aimed to de-emphasize or deflect online communication that associates sexual activity with the child.

Because many mothers felt they needed more time to consider the vaccine, they suggested providers should introduce the topic of HPV vaccination as anticipatory guidance, prior to the intended date for which the adolescent is targeted to receive the vaccine. For example: “*Often I don’t get info until day of shots. It may not be an easy decision unless they can get that information beforehand. They should have a list of pre-questions to ask and be prepared with questions to come in. They need information more than five minutes before they have to have it”* (Participant 15). The most common time frame suggested was 1 year in advance; others recommended discussion at age 8 or 9.

## Discussion

Parental hesitancy is considered one of the strongest reasons for low HPV vaccination coverage among U.S. adolescents, resulting in missed opportunities for cancer prevention [5]. Indeed, in a recent qualitative study of 43 office visits, Shay and colleagues found that parents in 37 of the 43 visits expressed hesitancy about the vaccine upon recommendation [30]. Although behavioral correlates of parental HPV vaccination decisions have been identified (e.g., prior influenza vaccination of the child) [31], provider recommendation is a consistently strong predictor; thus, to prevent HPV-related cancers, providers must communicate effectively with hesitant parents. As this study shows, erroneous and misleading HPV vaccine information in online media, social media and commercials, in particular, are sources of hesitancy that providers must address in communications.

Voices of mothers who are HPV vaccine-accepting but hesitant were uniquely analyzed in this study to show 1) how hesitancy can exist even amid acceptance, and 2) to highlight how exposure to commercial advertising, online media and social media use prior to the provider visit interfere with important HPV vaccination decisions about topics of safety, efficacy and sexual stigma. Online information about vaccines is ubiquitous, and research shows that when individuals encounter a large amount of health information online, it can lead to doubt and unnecessary anxiety and fear [32–34], which subsequently can lead to negative attitudes and behaviors toward a particular health issue [34]. These study results indicated that mothers had difficulty confidently accepting providers’ HPV recommendation over the many misleading messages about HPV vaccination in the media, leading to hesitancy and delay. Other studies have also found that contradictory and misleading information about vaccines has led parents to question the safety of childhood vaccination and contributed to the problem of vaccine hesitancy [35, 36]. These results and others indicate that providers must clearly and strongly, but sensitively [11, 14], communicate that the vaccine prevents cancer in order to overcome negative fear messages.

As a result of the hesitancy mothers felt from misinformation in the media, mothers suggested strategies for HPV vaccine recommendation communication that may help providers deliver recommendations to facilitate confident acceptance. However, many of the recommended strategies for improving the context and delivery of provider HPV vaccine recommendation, specifically those related to the timing of delivery and participatory approach, are somewhat at odds with the presumptive recommendation approach, which is the consensus preferred communication strategy [37]. While abandoning the presumptive recommendation approach is not suggested, providers might consider enhancing the approach by ensuring that mothers do not feel pressured into vaccination and by including upcoming HPV vaccination as part of the anticipatory guidance process. Having providers introduce the vaccine at an early age, along with helping mothers to access accurate and credible HPV vaccine information, might encourage greater confidence and acceptance of HPV vaccination.

Furthermore, vaccine hesitancy should not necessarily be viewed as an unchangeable belief. For example, Kornides and colleagues found that secondary acceptance of HPV vaccination is common, with more than two-thirds of parents in a national online survey accepting or intending to accept HPV vaccination after original refusal [38]. They recommend that providers seek to motivate secondary acceptance by delivering repeated, high-quality recommendations for HPV vaccination [38].

Confidence boosting and hesitancy reduction strategies may be needed to encourage hesitant, but vaccinating parents to complete the HPV vaccine series and to vaccinate younger children. Unaddressed concerns, particularly those that arise from negative media messages, may lead to increases in hesitancy over time. Additionally, vaccinating mothers who are not confident in their decision are unlikely to serve as strong advocates for HPV vaccination to other parents. In order to counter the false HPV vaccine narratives in the media, providers may need to ensure that they provide mothers with credible, timely information sources, thereby helping parents feel confident in their decision and, perhaps, more willing to vocally support HPV vaccination in their social network.

### Limitations and future direction

While the qualitative study design allowed for in-depth examination of the topic, there are limitations. Although a sample size of 30 interviews was adequate for this qualitative study, it limits the generalizability of findings, which may not apply to different groups of mothers and geographical locations. The mothers included in this study were highly educated and wanted more information about the HPV vaccine, which may not be true of other groups of mothers. Further, fathers were not interviewed and may have different media consumption patterns that reflect differently on the HPV vaccination decisional process and should thus be studied for comparison. Another future research direction would be to do a comparative study regarding media consumption of mothers who reject HPV vaccination and those who accept vaccination. Additionally, while qualitative research allowed for exploration of attitudes and behaviors surrounding the subject, media causation of hesitancy cannot be claimed. Quantitative approaches are recommended to examine the predictive nature of media information on HPV vaccination hesitancy.

## Conclusions

Results indicate that educated mothers who accept HPV vaccination for their children may do so with hesitancy due to misperceptions stemming from online media, social media and commercials. Given that media information about the HPV vaccine can be inaccurate and anxiety-producing, providers need to understand how media consumption may influence the way mothers experience and respond to an HPV vaccine recommendation. Providers should be prepared to address maternal fears and misunderstandings regarding safety, protection and sexual stigma. A provider’s recommendation style and technique must also include preparation to counteract negative media messages in order to reduce HPV vaccination delays and hesitancy.

## Supplementary Information

**Additional file 1:** **Appendix A.** Interview guide.

## Data Availability

The datasets used and/or analysed during the current study are available from the corresponding author on reasonable request.
