# Supplementary material for: The role of the media on maternal confidence in provider HPV recommendation
Source: BMC Public Health. 2020 Nov 23;20:1765. doi: 10.1186/s12889-020-09877-x (PMC7684709; doi:10.1186/s12889-020-09877-x)
Supplement: Supplementary file 1 — Additional file 1: Appendix A. Interview guide. [file 12889_2020_9877_MOESM1_ESM.docx]

| **Appendix A.** Interview guide |
| --- |
| 1. How did you come to a decision to vaccinate your child for HPV? |
| 1A. General probing questions: What was important to you in the decision? Who or what did you look for to trust your decision? Did you vaccinate immediately upon vaccination; if not, what did you do?  1B. Media probe: Do you recall anything that you read, heard or saw in the media at the time of provider recommendation? Did the information cause hesitancy to vaccinate?  How did it influence your ultimate decision to vaccinate? |
| 1. Please describe your experience and what you remember about your decision about the HPV vaccine for your child?   2A. Media probe: How did the media (what you read, saw, heard) influence your  decision about the HPV vaccine for your child, if at all?  2B. Media probe: Describe the specific information you recall thinking about when  deciding about the HPV vaccine for your child (upon provider recommendation)?  2C. Media probe: Describe how the information you recalled in the media impacted your  decision about the HPV vaccine for your child? |
| 1. What were the concerns you had, if any, about deciding to vaccinate your child (e.g   or following your providers’ recommendation to vaccinate)?  3A. Describe the specific concerns you had that stemmed from the media that caused  hesitancy accepting your provider’s recommendation? |
| 4. Where did the concerns stem from; what were the sources of the concerns?  4A. Media probe: Where did you read, see or hear in the media the HPV vaccines you described ((e.g. What were the sources of media/channels)? |
| 1. Was your decision about the HPV vaccine and its recommendation different or   similar to other vaccines and provider recommendation for them? Why or why not?  4A. Media probe: What do you recall reading, seeing or hearing in the media that made  you hesitate or believe that the HPV vaccine recommendation was different than others? |
| 1. What do you think other mothers’ thoughts about the HPV vaccination are, including   any barriers they may have about HPV vaccination for their child?  5A. Media probe: What do you think other mothers’ thoughts about the HPV vaccine are  from what you believe they, too, read, see and hear in the media? |
| 1. What can providers do to help empower mothers to vaccinate their child for HPV?   6A. Media probe: What can providers do to help mothers overcome negative media  influences to help them feel more confident about accepting HPV vaccination? |
